# The expression of ecdysteroid UDP-glucosyltransferase enhances cocoon shell ratio by reducing ecdysteroid titre in last-instar larvae of silkworm, *Bombyx mori*

**DOI:** 10.1038/s41598-018-36261-y

**Published:** 2018-12-07

**Authors:** Guanwang Shen, Jinxin Wu, Yong Wang, Hongling Liu, Haiyan Zhang, Sanyuan Ma, Chuyue Peng, Ying Lin, Qingyou Xia

**Affiliations:** 1 State Key Laboratory of Silkworm Genome Biology, Southwest University, Chongqing, 400716 China; 2 Chongqing Engineering and Technology Research Center for Novel Silk Materials, Chongqing, 400716 China; 3 College of Biotechnology in Southwest University, Chongqing, 400716 China

## Abstract

Ecdysteroid UDP glucosyltransferase (EGT) is a baculovirus-encoded protein which can hinder the normal molting of insects by inactivating 20-hydroxyecdysone (20E). Here we expressed EGT in the last-instar silkworm larvae using the GAL4/ UAS system. Compared with the control, for the EGT overexpressed silkworm, the hemolymph 20E content was significantly decreased, the feeding and spinning periods of the last-instar silkworm larvae were extended, the cocoon shell ratio was significantly increased, and the transformation from silkworm larvae to pupa was blocked. Increasing EGT expression resulted in the decrease of 20E content in the hemolymph of silkworm larvae, treating the EGT overexpressed male silkworm with 20E decreased the larval weight and cocoon shell ratio, confirming that the increase in the availability of nutrients to the cocoon and an increase in the cocoon shell weight in the hybrid transgenic silkworms is because of the EGT-induced reduction in active 20E content. Furthermore, though the sericin and flavonoid contents were increased in the cocoon of the EGT overexpressing silkworm, the production of silk fibroin didn’t change.

## Introduction

The silkworm (*Bombyx mori)* is a representative member of the order Lepidoptera. It has many advantages as a model organism, including availability of the complete genome sequence^1,2^, short life cycle, moderate size, and easy cultivation^3–6^. Thus, the silkworm has been widely used in molecular biology, physiology, developmental, toxicology, and medical studies. It has four distinct developmental stages including egg, larva, pupa, and moth^7^. Larvae undergo several rounds of moulting, during which nutrients taken up from mulberry leaves are used to construct the cocoon and prepare for pupal development and adult organ formation. The other three developmental stages mainly serve to allocate nutrients accumulated in larvae.

Pupae are the intermediate stage in the metamorphosis from larva to adult. The transition from late last-instar larva to early pupa is the most important period for nutrient distribution, as larvae stop feeding and start spinning a protective cocoon. The allocation of nutrients for this process is under strict genetic and hormonal control. Ecdysone (20-hydroxyecdysone, 20E) regulates the development of the pre-pupa^8^; the level is relatively low in the spinning stage and increases after spinning^9,10^. A high concentration of 20E in the haemolymph promotes the degradation of larval tissues including silk glands^11–13^, leading to pupation^14,15^. However, it is unclear whether 20E affects the absorption and utilization of nutrients in silk production. If so, modulating 20E levels could delay pupation and prolong the spinning stage, leading to increased silk production.

Ecdysteroid UDP glucosyltransferase (EGT) is a baculovirus-encoded protein^16,17^ that is secreted by *Acanthopanax* sp. heterozygous nucleopolyhedrovirus (NPV) and transfers UDP-glucoside to the hydroxyl group of carbon 22 in 20E to generate 20E 22-β-d-pyran glucoside^18,19^. The EGT secreted by the virus upon infection can inactivate 20E in the host, thereby hindering normal moulting and eclosion^20–22^. Transient expression of the *EGT* gene of *B. mori* NPV in silkworm pupae was shown to prevent the emergence of silkworms and prolong the pupal stage^23^. Thus, overexpressing EGT to reduce the content of active 20E in the haemolymph of late last-instar larvae can affect the progression of silkworm metamorphosis. We previously reported that a fragment of the *B. mori 30* *K lipoprotein* (*BmLP3*) promoter expressed exclusively in the fat body of the late last-instar larval to early pupal stage could drive the expression of an exogenous protein with the same temporal and spatial distribution as endogenous LP3^24,25^.

The GAL4/upstream activating sequence (UAS) expression system has been used in various model organisms including *Arabidopsis thaliana*^26^, *Mus musculus*^27^, *Danio rerio*^28,29^, and *Drosophila melanogaster*^30^. It has also been used to generate transgenic silkworms^31,32^ and expressing scorpion venom at the pupal stage^33^. Although EGT was shown to reduce the concentration of active 20E in silkworms, the toxic and lethal effects of EGT could affect productivity^23^. In the present study, we used GAL4/UAS system to express EGT from the *LP3* promoter in the silkworm fat body of last-instar larvae. The EGT protein secreted into the silkworm haemolymph maintained 20E at a low level, allowing us to investigate the role of 20E in the utilization of available nutrients for silk production.

## Results

### Generation of transgenic silkworms

**Figure 1:**
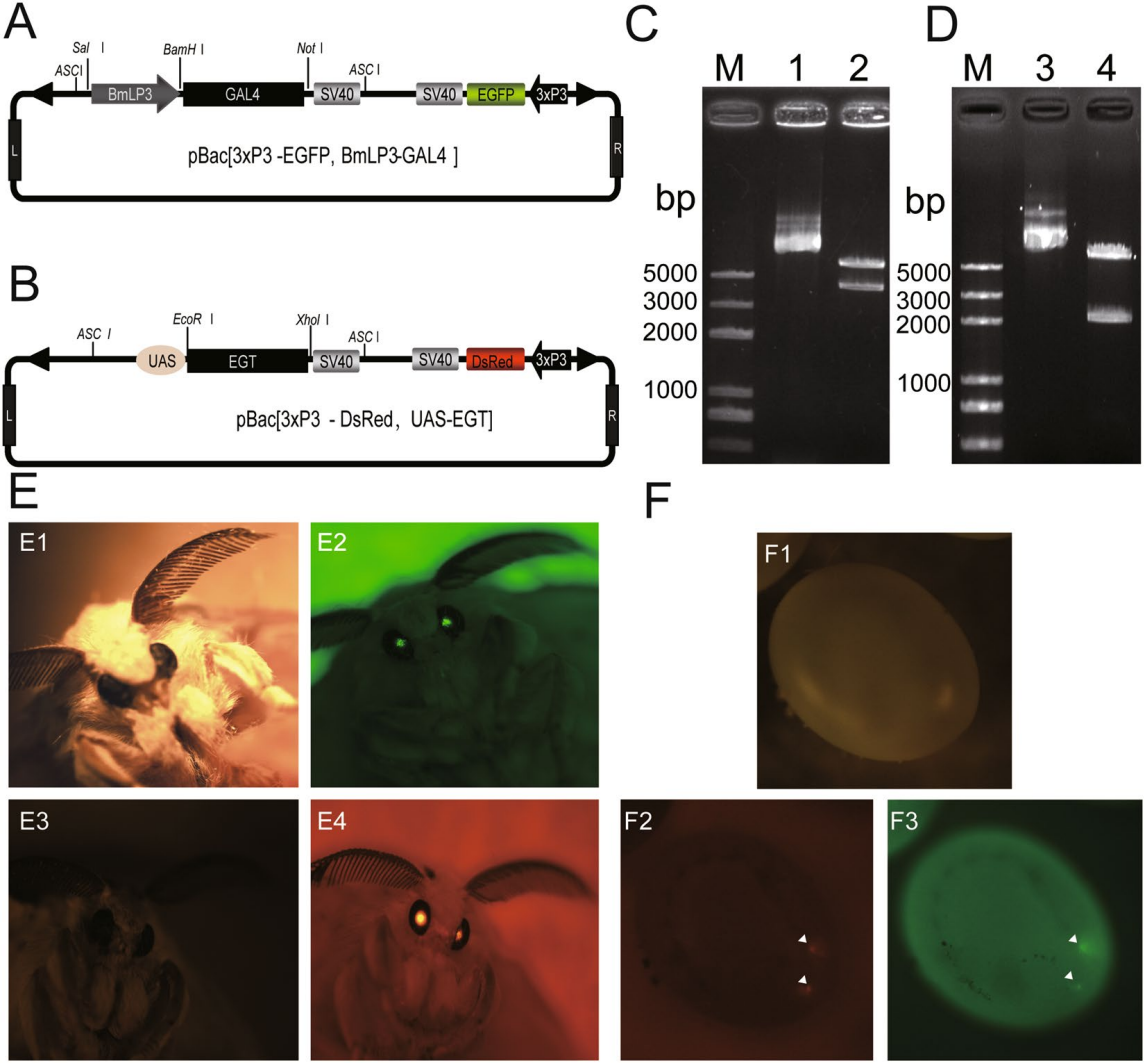
Isolation of transgenic silkworms. (**A**,**B)** Schematic illustration of the piggyBac [3 × P3-EGFP, BmLP3-Gal4] and piggyBac [3 × P3-DsRed, UAS-EGT] vectors. (**C**,**D**) Digestion of the vectors with *Asc*I yielded the expected band sizes. Lane 1/3: agarose gel electrophoresis detection of piggyBac [3 × P3-EGFP, BmLP3-Gal4]/piggyBac [3 × P3-DsRed, UAS-EGT] vector plasmid; Lane 2/4: agarose gel electrophoresis detection of piggyBac [3 × P3-EGFP, BmLP3-Gal4]/piggyBac [3 × P3-DsRed, UAS-EGT] vector plasmid after *Asc*I digestion. BmLP3-Gal4-SV40 is about 4000 bp and UAS-EGT-SV40 is about 2100 bp. (**E**) Isolation of generation G0 transgenic silkworms by detection of EGFP (Gal4 transgenic line) and DsRed (UAS transgenic line) expression in the compound eyes of moths. E1/E3: Gal4/ UAS transgenic moth under white light. E2/E4: Gal4/UAS transgenic moth under fluorescent light. (**F**) Screening of generation G7 hybrid offspring from Gal4 and UAS transgenic lines by detection of both EGFP and DsRed expression in the eggs. F1/2/3: transgenic eggs under white/green fluorescent/red fluorescent light.

We used EGT to reduce 20E content at the late last-instar larval stage by constructing two activator vectors, piggyBac [3 × P3-EGFP, BmLP3-Gal4] (Fig. 1A) and piggyBac [3 × P3-DsRed, UAS-EGT] (Fig. 1B). Digestion of the plasmids with *Asc*I yielded bands of the expected sizes (Fig. 1C,D).

After injecting the transgenic vectors into the eggs of silkworms, green/red fluorescence was detected in the compound eyes of generation G0 moths (Fig. 1E). Generation G7 of the BmLP3-Gal4 line was crossed with the G7 UAS-EGT transgenic line and the eggs laid by these moths showed both red and green fluorescence (Fig. 1F) indicating that the piggyBac vectors were inserted into the silkworm genome.

### Last-instar larvae development and increased availability of nutrients in transgenic silkworms

**Figure 2:**
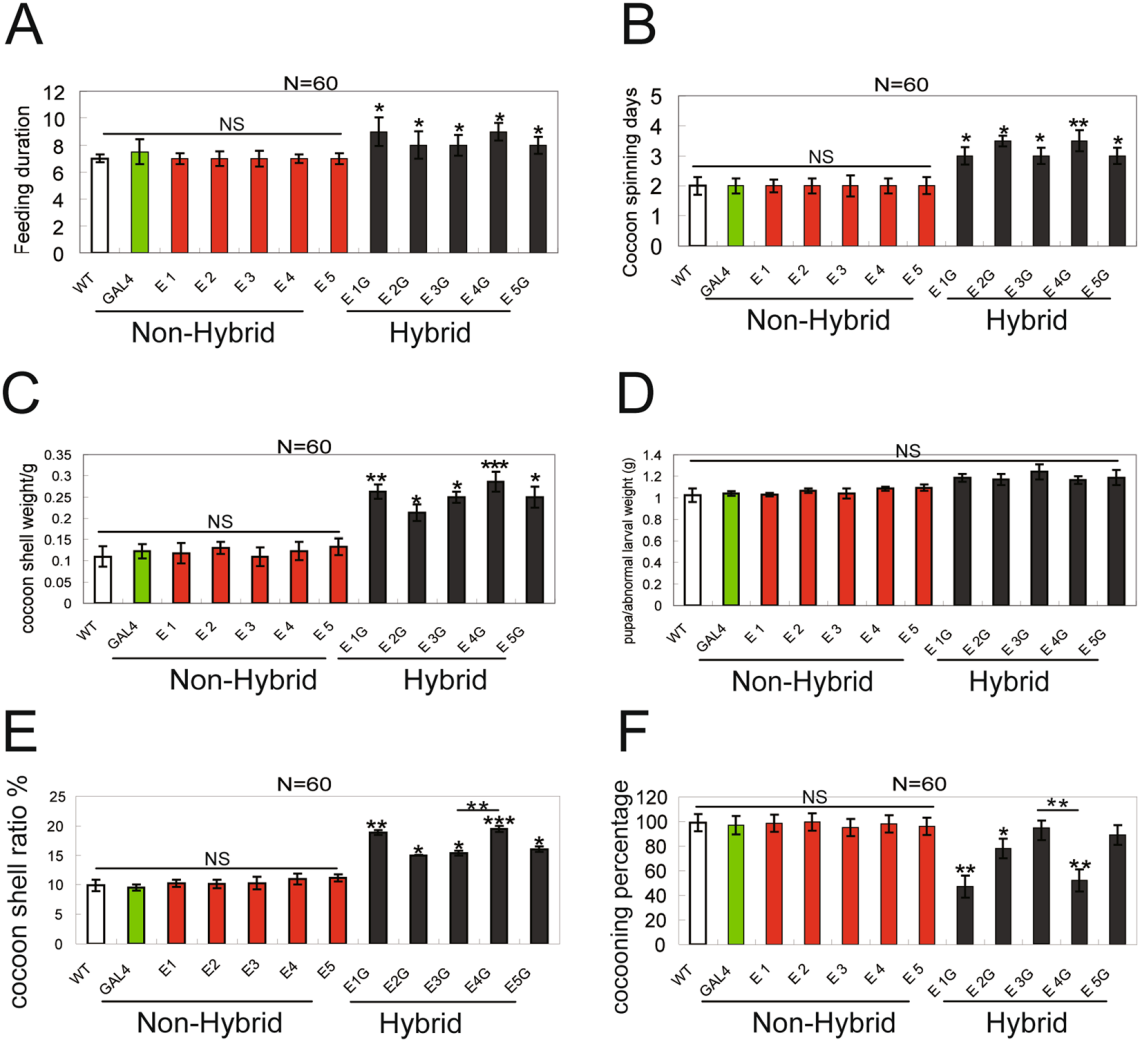
Last-instar larvae development. (**A**,**B**) Duration of feeding (**A**) and cocoon spinning (**B**) stages. (**C**) Cocoon shell weight. (**D**) Pupa/abnormal larval weight. (**E**) Cocoon shell ratio (in percentage). (**F**) Cocooning percentage.

We generated one Gal4 and five UAS-EGT transgenic lines that were reared on mulberry leaves up to generation G7, before intercrossing. The development and survival of hybrid offspring before the last-instar larval stage did not differ from the non-transgenic control (Fig. S1A/B). The number of feeding (Fig. 2A) and spinning (Fig. 2B) days and silk production (cocoon shell weight and ratio; Fig. 2C,E, respectively) of hybrid offspring at the last-instar larval stage were higher than those in non-transgenic or non-hybrid offspring. The cocooning percentage showed a decrease except for E3G and E5G lines (Fig. 2F), but the pupa/abnormal larvae weight showed no significant difference at cocoon spinning three days after (Fig. 2D). These results demonstrate that the extension of larval development leads to the increased amount of some silk proteins, although we did not observe any effect on silk fibroin.

### EGT expression and function in transgenic silkworms

**Figure 3:**
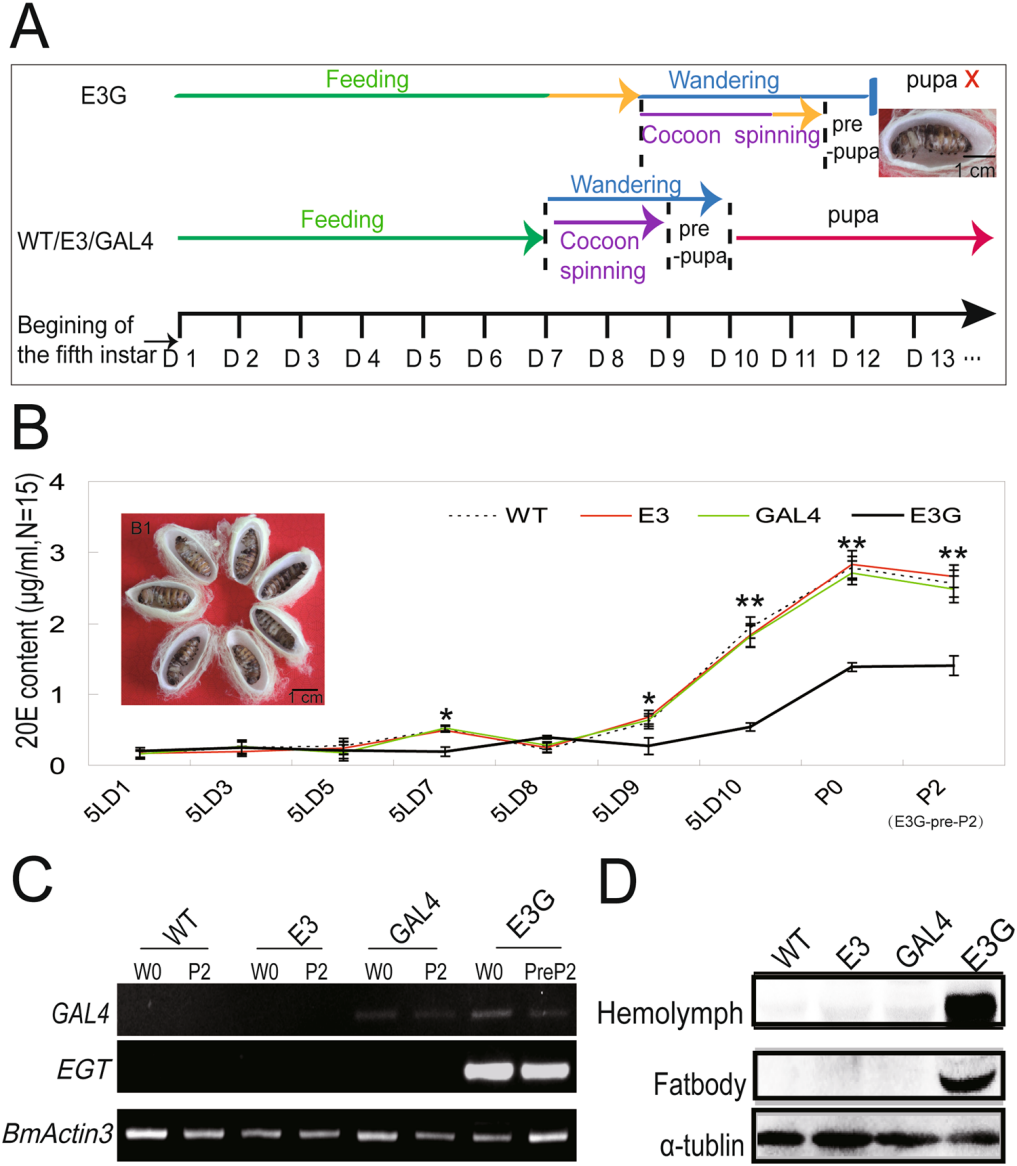
Phenotype, 20E content, and EGT expression of hybrid transgenic silkworm larvae (E3G). (**A**) Time point of developmental state among different silkworm strains. (**B**) Detection of 20E content in the haemolymph of last-instar larvae by ELISA. B_1_ Phenotype of E3G larvae on day 7 after wandering. 5LD1/3/5/7/8/9/10 indicate day 1/3/5/7/8/9/10 of fifth-instar larvae. (**C**) GAL4 and EGT mRNA levels in last-instar larvae fat bodies at 0 h after wandering (W0) and day 2 of pupa/prepupa (P2/PreP2). (**D**) EGT protein level of last-instar larvae on day 3 after wandering.

The hybrid offspring E3G cocoon shell ratio was increased (Fig. 2E) while cocooning percentage was unaltered (Fig. 2F). However, according to statistics of developmental time point in different strains, E3G silkworm larvae have a longer feeding and cocoon spinning stage and stay in pre-pupa that cannot pupate (Fig. 3A). In addition, the development of E3G was stopped between the larval and pupal stages, indicating that metamorphosis was blocked between these stages (Fig. 3B1). To confirm that the observed phenotypic effects resulted from the change of 20E content, enzyme-linked immunosorbent assay (ELISA) was performed to measure 20E level in last-instar larvae. The data revealed that the content of 20E in the haemolymph was lower in hybrid silkworms on days 7, 9, and 10 of the last-instar larval stage than in the control group. The control group 20E titers reached a peak at 5LD7, while 20E titer of E3G reached a relatively small peak at 5LD8. So the appearance of the peak of 20E titers was delayed for about 1 day in E3G larvae (Fig. 3B), consistent with the prolonged feeding duration of the hybrid transgenic silkworm strains.

To further investigate if the decreased 20E content resulted from EGT expression, RNA was extracted from the fat body of last-instar larvae at feeding cessation (W0, spinning starts, larvae enter wandering stage) and day 2 of pupal stage (P2: non-hybrid silkworms, Pre-P2: hybrid silkworms-E3G). Reverse transcription PCR showed that Gal4 was expressed in GAL4 and E3G hybrid lines but not in E3, whereas EGT was strongly expressed in E3G only (Figs 3C and S3A). In addition, we were able to detect EGT protein in silkworm’s haemolymph and fat body (Figs 3D and S3B). This indicated that the Gal4/UAS system was functional and that EGT over-expression in last-instar larvae reduced the 20E content in the haemolymph.

### Cocoon composition in transgenic silkworms

**Figure 4:**
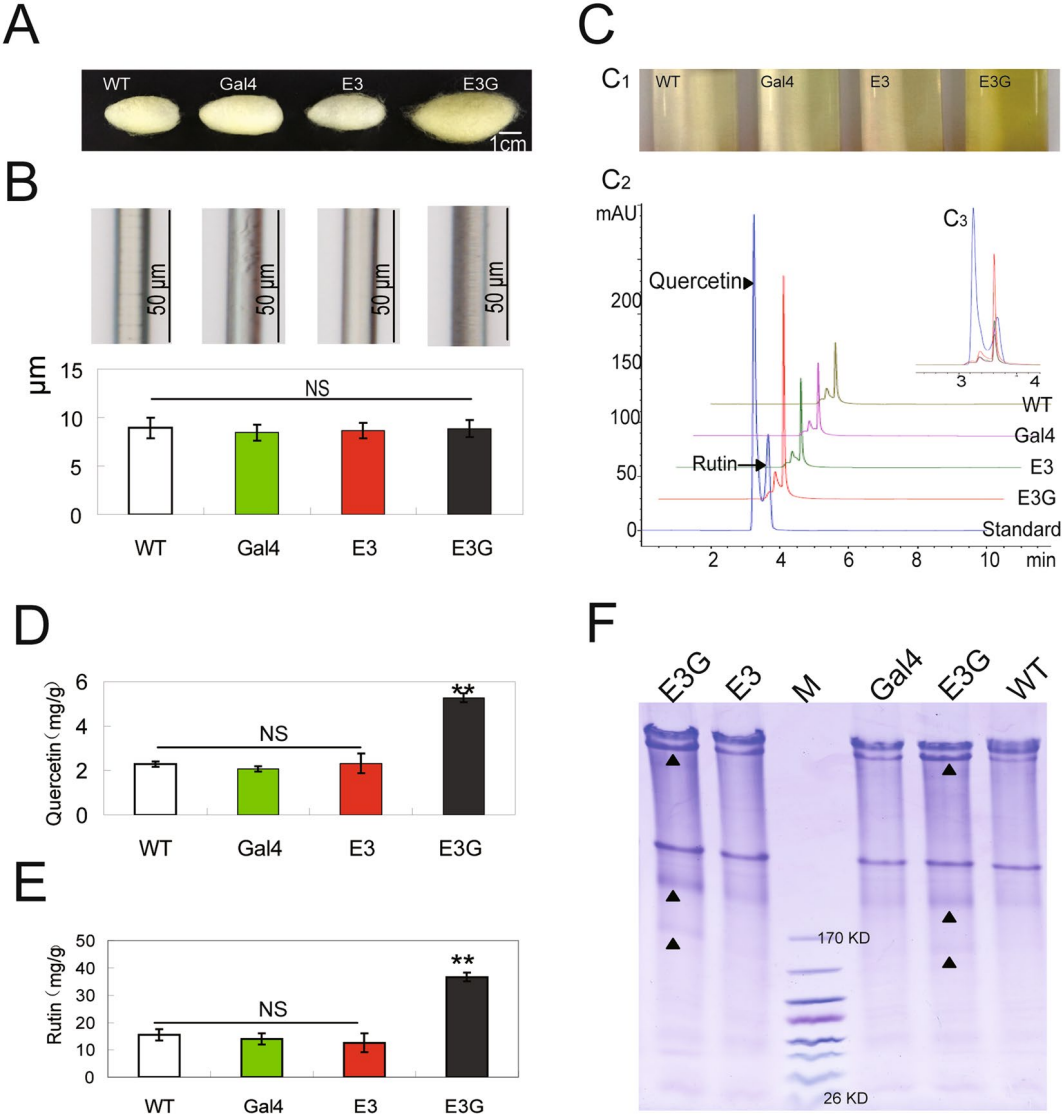
Detection of flavonoid and total sericin in the cocoon of transgenic silkworm. (**A**) Phenotype of cocoons. (**B**) Diameter of silk fibres produced by wandering-stage silkworms. (**C**) Detection of compounds in the cocoon by HPLC (C_1_, flavonoid extract solution; C_2_, analysis of flavonoid extract solution by HPLC; C_3_, overlapping peaks in the chromatogram). (**D**,**E**) Quantitative analysis of quercetin/rutin content in cocoons by HPLC. (**F**) Analysis of total sericin content in cocoons by SDS-PAGE.

Since the cocoons of the hybrid silkworm were bigger than that of the non-hybrid silkworms (Fig. 4A), we examined the characteristics of the cocoon by artificially reeling the silk from eight silkworms at the beginning of wandering. Optical microscope analysis revealed no differences in fibre diameter (Fig. 4B).

The cocoon is mainly composed of flavonoids, small molecules, and silk protein. We examined whether the increase in nutrient allocation to the cocoon affected its molecular composition and found that the solution obtained by dissolving hybrid transgenic silkworm cocoon was more yellow in colour than that of the control (Fig. 4C1). Two flavonoids were detected in the solution by high-performance liquid chromatography (HPLC; Fig. 4C2); these were identified as quercetin (Fig. 4D) and rutin (Fig. 4E). Further, to analyse the sericin content, the cocoon was cut into small pieces and degassed with an 8 M urea solution at 100 °C for 30 minutes. The supernatant was collected by centrifugation and used for sodium dodecyl sulphate polyacrylamide gel electrophoresis (SDS-PAGE). Results showed that sericin protein content (extracted from silkworm cocoons with the same weight in each group) was higher in the cocoon of hybrid transgenic silkworms than in the control group, especially at the position indicated by the black arrow in Fig. 4F. Thus, the decrease in 20E level resulted in flavonoid and sericin accumulation in the cocoon.

**Figure 5:**
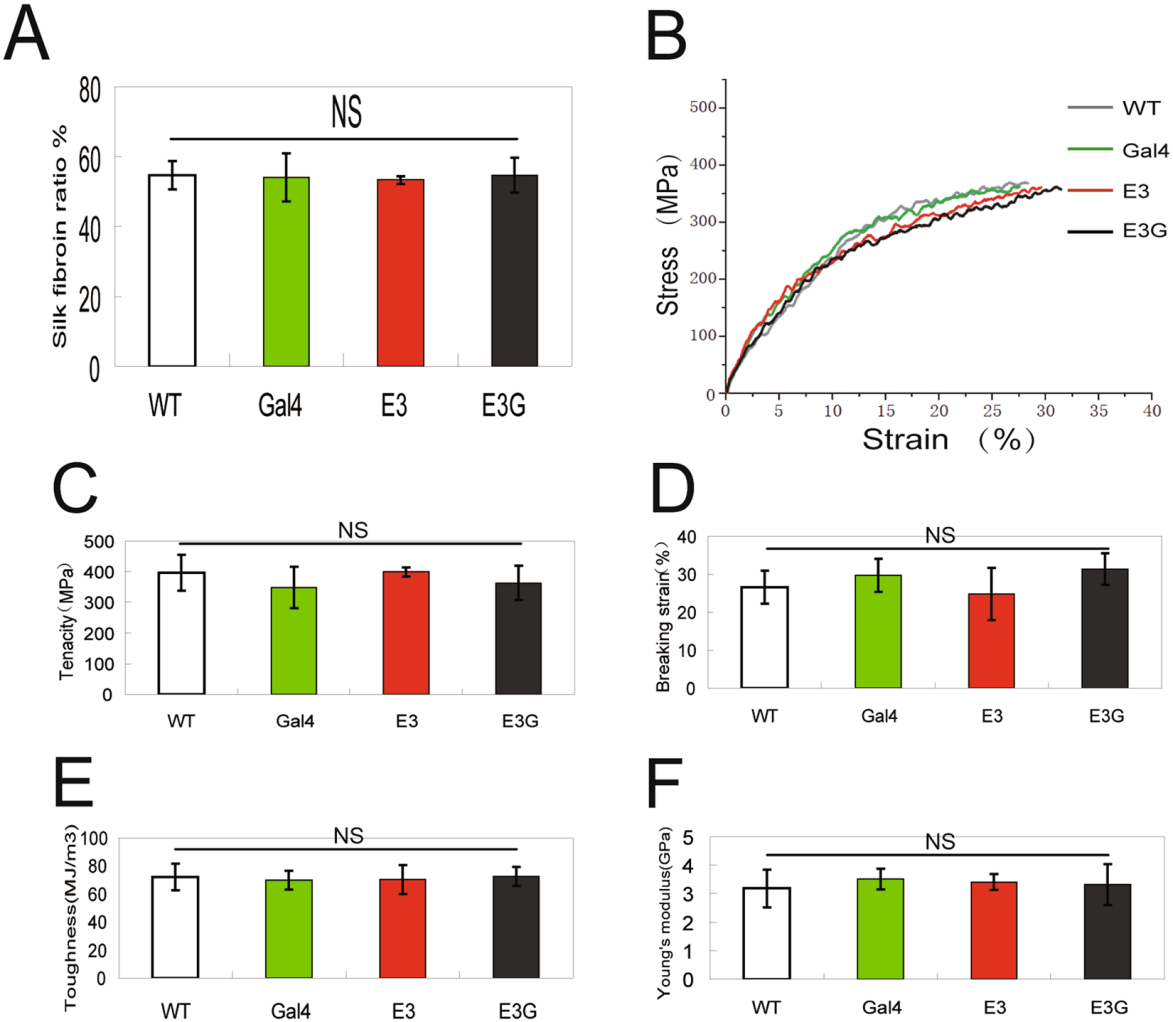
Mechanical properties of silk fibroin in transgenic silkworm. (**A**) Proportion of silk fibroin in the cocoon. (**B**) Stress-strain curves for silk fibroin. The curves represent an average of 30 experiments per group. (**C**–**F**) Tenacity (**C**), breaking strain (**D**), toughness (**E**), and Young’s modulus (**F**) of silk fibroin obtained from wandering-stage silkworms.

**Figure 6:**
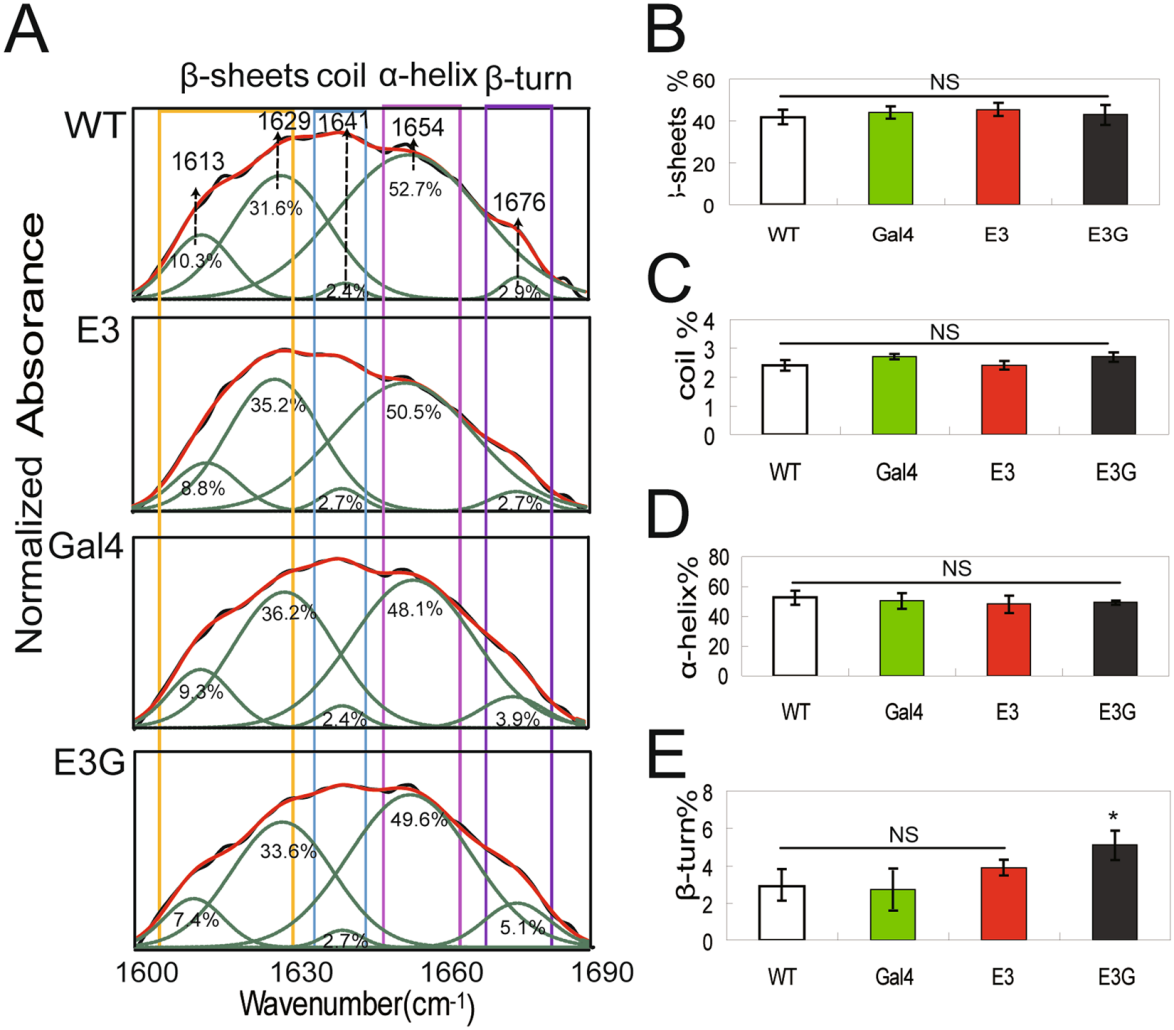
FTIR microspectroscopy analysis of silk fibroin secondary structure. (**A**) Curve fitting of the amide I region of silk fibroin in wandering-stage silkworms. (**B**–**E**) Proportion of β-sheets (**B**), random coils (**C**), α-helices (**D**), and β-turns (**E**) in silk fibroin obtained from wandering-stage silkworms.

After removing sericin and small molecules, the cocoon contained only silk fibroin and its ratio (fibroin air-dried weight/cocoon shell weight × 100%) in hybrid transgenic silkworm did not differ from that of control silkworm (Fig. 5A). Mechanical testing of silk fibroin revealed no differences in the stress–strain curves (Fig. 5B) or in average tenacity (Fig. 5C), breaking strain (Fig. 5D), toughness (Fig. 5E), or Young’s modulus (Fig. 5F) between silk fibroin from non-transgenic and transgenic silkworms. Analysis of the secondary structure of silk fibroin by Fourier-transform infrared (FTIR) microspectroscopy (Fig. 6A) showed that the proportion of β-sheets (Fig. 6B), random coils (Fig. 6C), and α-helices (Fig. 6D) were similar, whereas the proportion of β-turns (Fig. 6E) was higher in hybrid transgenic silkworm than in non-transgenic silkworm. These results demonstrate that a reduction in 20E titre at the last-instar larval stage had no impact on the mechanical properties of silk fibroin, although sericin protein accumulation was increased.

### Effect of exogenous 20E on last-instar larvae development and cocoon formation

**Figure 7:**
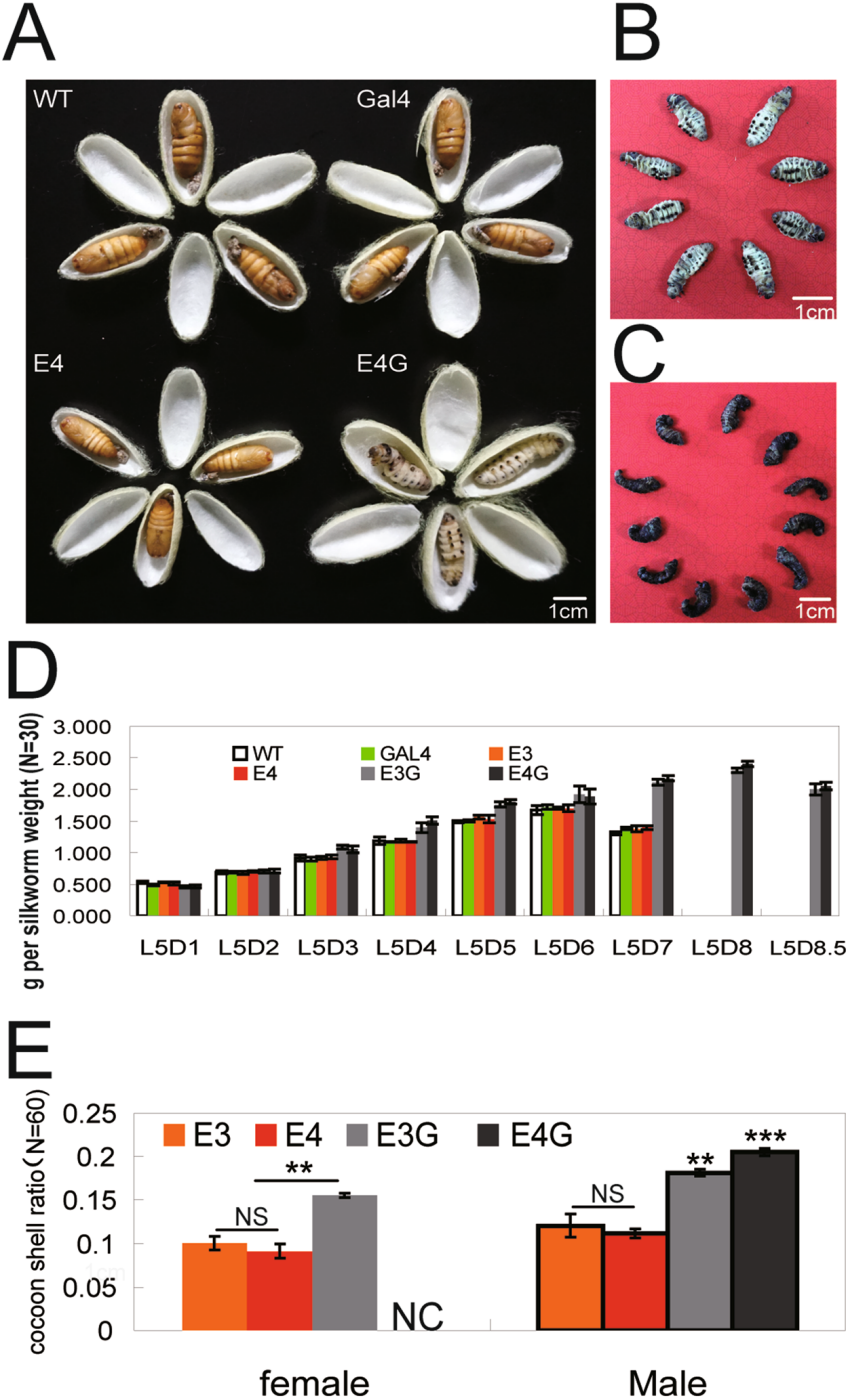
Last-instar larvae development in hybrid transgenic silkworm offspring. (**A**–**C**) Phenotype of transgenic hybrid silkworm E4G on days 7, 14, and 28 after wandering. (**D**) Weight and duration of feeding stage in last-instar larvae. (**E**) Cocoon shell ratios in male and female silkworms. NC indicates “No Cocoon”.

Cocoon shell ratio (Fig. 2E) and cocooning percentage (Fig. 2F) varied among hybrid offspring. The cocoon shell ratio of E4G was higher than that of E3G (Fig. 2E) and they ceased developing at an earlier time point (Figs 3A1 and 7A); however, cocooning percentage in E4G (Fig. 2F) was only half that of E3G. On the other hand, both E3G and E4G hybrid transgenic fifth-instar larvae fed for about 1.5 days more than non-hybrid transgenic and non-transgenic silkworms (Fig. 7D). Male fifth-instar larvae showed normal cocoon spinning after they stopped eating, and the cocoon shell ratio of E3G male and E4G male was higher than that of non-hybrid transgenic E3 and E4. However, female E4G fifth-instar larvae showed almost no cocoon spinning until death, after they stopped feeding (Fig. 7E). Thus, the low cocooning percentage of E4G was due to cocooning failure in E4G females.

**Figure 8:**
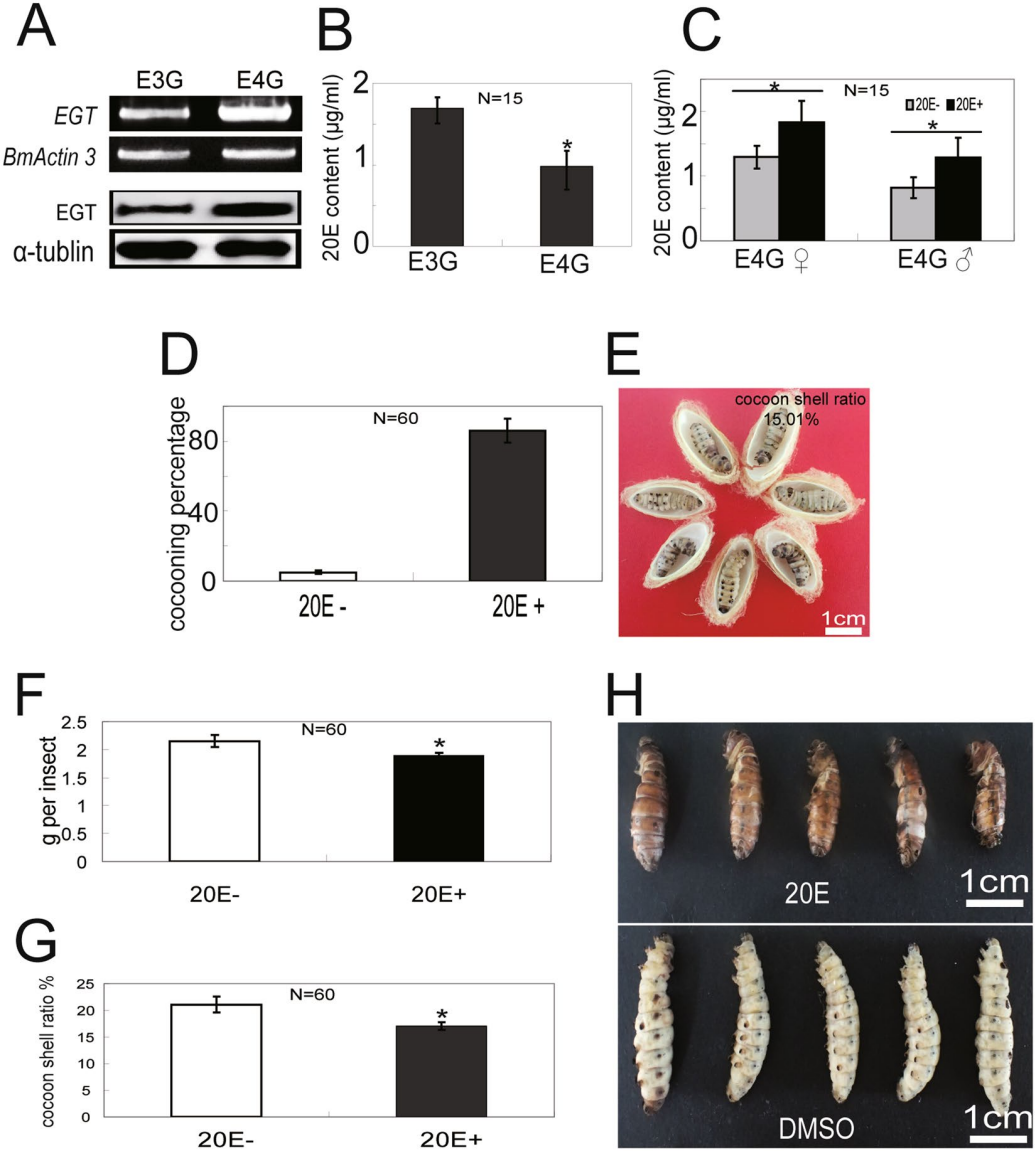
EGT expression and 20E content in E3G vs. E4G larvae and the effect of 20E administration on 20E content and larval development. (**A**,**B**) EGT expression level (**A**) and 20E content (**B**) in E3G and E4G last-instar larvae 120 h after wandering. (**C**) Hemolymph 20E content in E4G female and male last-instar larvae 5 days after feeding on 20E-treated mulberry leaves. (**D**) E4G female cocooning percentage. (**E**) E4G female phenotype after 20E administration. (**F**) Larval weight of E4G W0 males. (**G**) Cocoon shell ratio of E4G males (in percentage). (**H**) E4G male phenotype after 20E administration.

To examine the relationship between EGT expression level and 20E content with the development of E3G/E4G, we compared EGT expression levels in E3G and E4G silkworms. The EGT transcript level in the fat body and EGT protein secretion into the haemolymph were both higher in E4G than in E3G (Fig. 8A). In addition, haemolymph 20E content was lower in E4G than in E3G (Fig. 8B). Increased content of 20E after its administration through feeding (Fig. 8C) promoted cocoon spinning in female E4G silkworms (Fig. 8D) but decreased W0 larval weight (Fig. 8F) and cocoon shell ratio in males (Fig. 8G), although development was enhanced in both female (Fig. 8E) and male (Fig. 8H) silkworms. Thus, EGT over-expression in last-instar larvae decreases 20E content in the haemolymph, thereby leading to the extension of feeding and spinning periods and blocking pupation, which increases the availability of nutrients to the cocoon.

## Discussion

It has been reported that juvenile hormone can inhibit 20E secretion during the early days of *B. mori* last (fifth)-instar larvae^34^, and extend the duration of feeding and silk yields^35,36^. Our study found that controlling 20E concentration via ectopic expression of EGT in the last larval instar can influence the duration of the larval feeding stage and cocoon shell ratio.

In hybrid transgenic silkworms, a significant reduction of 20E content was observed only from day 7 of fifth larval instar. However, no significant difference of 20E concentration was observed from the early fifth instar to the fifth day of the fifth larval instar (5LD5) between different silkworms strains. This might be due to the fact that under the control of BmLP3, the larvae are expected to express the target gene only from day 4 of feeding in the fifth larval instar^21^. The silkworm last-instar larvae, after they stop feeding, empty their bowels and enter a period of maturation before starting to spin the cocoon. During this time, 20E titer shows a small increase^10,37,38^.

In the present study, compared with the control group, the peak of 20E titer in E3G larvae was delayed for about 1 day (5LD7 for control group vs. 5LD8 for E3G, Fig. 3B). A prolongation of feeding duration by almost 1.5 days in hybrid transgenic larvae E3G thus might be related with the early expression of EGT to reduce the 20E content resulting in a delayed peak of 20E and a delay in the wandering and the spinning behaviour of the hybrid transgenic last-instar larvae. Further, E4G hybrid silkworm larvae, which have significantly lower content of 20E and much higher expression of EGT than E3G larvae ceased developing at an earlier time point than E3G larvae (Figs 3B1, 7A and 8A,B). In addition, females E4G could not form cocoon but E3G hybrid transgenic females could. However, female E4G is able to form normal cocoon after 20E treatment. The present study suggests thus the role of 20E in promoting cocoon spinning as E4G females are able to form normal cocoons after 20E treatment.

E3G hybrid transgenic silkworms did not pupate normally after spinning and this could be due to the expression of EGT at the beginning of the cocooning and pre-pupal stage and a reduction of 20E content. Further, E4G larvae didn’t enter into the normal pupal developmental program and this could be due to a higher expression of EGT and a severe reduction in 20E content in these larvae, as the pupal developmental program in these larvae was partly rescued after treatment with 20E. Moreover, E3G transgenic silkworms, expressing a relatively low level of EGT and an increased content of 20E than E4G, were unable to survive but showed some phenotypic characteristics of the pupae. Cocoon shell ratio was also lower in these hybrids than in silkworms with high EGT expression (E4G). In E3G larvae, as the expression of EGT is relatively low, the inactivation of 20E is also at a minimum. Thus, these larvae showed a relatively higher 20E content than E4G larvae. Wang *et al*.^39^ showed that the treatment of 20E decreases the consumption of food in silkworm larvae. Thus, a relatively greater level of 20E in E3G larvae might have decreased the consumption of food and the availability of nutrients towards cocoons formation, resulting in lower cocoon shell ratio when compared with E4G. Further, the treatment of hybrid transgenic male E4G silkworm larvae with 20E decreased the larval weight and cocoon shell ratio, confirming that the increase in the availability of nutrients to the cocoon and an increase in the cocoon shell weight in the hybrid transgenic silkworms is because of the EGT-induced reduction in active 20E content.

Flavonoid content was increased in the cocoons of hybrid transgenic silkworms, and this is likely to be related to the homology between EGT and other endogenous enzymes (e.g. uridine diphosphate glucosyl transferase, NP-001135960.1) that catalyse flavonoid formation (Fig S2). We cannot exclude that the EGT would have an impact on some other enzymes, however, it seems more likely that the extension of the last larval instar causes the production of more flavonoids. Here, sericin content was increased in hybrid transgenic silkworms compared to non-transgenic silkworm; thus, the role of 20E in regulating the expression of silk protein genes warrants further investigation.

Silkworm is economically important due to its unique ability to spin silk. As such, a major concern in sericulture is to distribute most nutrients accumulated in larvae towards cocoon spinning to increase silk yield. Silkworm generated by traditional breeding methods has reached its upper limit for silk yield, and this can only be further improved using molecular biology-based approaches. Here, we developed a transgenic silkworm line in which EGT is expressed at the last-instar larval stage using the GAL4/UAS system. Overexpression of EGT decreased the content of active 20E in the haemolymph and blocked the development after cocoon spinning. Because pierced cocoons are unsuitable for reeling, pupae are killed by heating before eclosion, and silk is reeled from dried cocoons. However, heating/drying cocoons is a labour-intensive and energy-consuming process. In the present study, we generated transgenic silkworms that were developmentally arrested after spinning, which eliminates the labour required for fresh cocoon processing and drying and saves the energy required for drying. Thus, expression of EGT at the last-instar larval stage using the GAL4/UAS system is a reliable approach for industrial applications and saves the energy required for drying.

## Methods

### Construction of piggyBac transgenic vectors

Genomic DNA was isolated from *B. mori* strain Dazao and used to amplify the *BmLP3* gene promoter fragment by PCR. The *Gal4* gene was amplified from the pSL[A4-GAL4] plasmid, which was constructed and stored in our laboratory. The following PCR profile was used to amplify target sequences: 95 °C for 5 min; 35 cycles of 95 °C for 30 s, 55 °C for 30 s, 72 °C for 1 min; and 72 °C for 10 min. Amplicons were gel purified and cloned into the pMD19-T Simple plasmid (Takara Bio, Otsu, Japan). The recombinant plasmid was confirmed by sequencing. The *EGT* gene sequence containing an upstream activation sequence (UAS) with *Asc*I and *Xho*I sites was synthesized by Takara Bio Corporation and cloned into the pMD19-T Simple plasmid to generate pMD19-T [UAS-EGT].

The *piggyBac* vector containing 3 × P3-EGFP can be used to screen transgenic silkworms. We constructed a *piggyBac* vector expressing the *Gal4* gene under the control of the *BmLP3* promoter as follows. The sequences were assembled into the intermediate plasmid pSL1180 (modified and stored in our laboratory) to generate a functional cassette, which was digested with *Asc*I and ligated into *piggyBac* [3 × P3-EGFP] using T4 DNA ligase (New England Biolabs, Ipswich, MA, USA), yielding *piggyBac* [3 × P3-EGFP, Bm LP3-Gal4].

Similarly, a *piggyBac* vector with 3 × P3-DsRed as a screening marker was prepared as follows. The *Asc*I–*Xho*I fragment of the PCR fragment from pMD19-T [UAS-EGT] was inserted into the *Asc*I–*Xho*I site of pSL1180 to generate the intermediate plasmid pSL1180 [UAS-EGT]. The cassette was excised and inserted into *piggyBac* [3 × P3-EGFP] to generate *piggyBac* [3 × P3-DsRed, UAS-EGT].

### Generation of transgenic silkworms

Transgenic silkworms were generated as previously described^40^ using the plasmids *piggyBac* [3 × P3-EGFP, Bm LP3-Gal4] and *piggyBac* [3 × P3-DsRed, UAS-EGT]. Generation G0 moths were screened for the expression of EGFP or DsRed in their compound eyes under an epifluorescence stereomicroscope (Olympus, Tokyo, Japan). The EGFP-/DsRed-positive individuals were mated with siblings and the offspring were raised for seven generations and maintained at the State Key Laboratory of Silkworm Genome Biology of Southwest University, Chongqing, China.

### Hybrid descendants of Gal4 and UAS transgenic silkworms

Generations G7 of BmLP3-Gal4 and UAS-EGT transgenic lines were crossed, and the hybrid offspring were reared on mulberry leaves. Data on feeding and wandering days of last-instar larvae, cocoon shell ratio (cocoon shell weight/whole cocoon weight × 100), cocooning percentage (total number of cocoons/total number of matured larvae × 100), and survival rate were recorded.

### FTIR microspectroscopy and analysis of the mechanical properties of silk fibers

At the beginning of cocoon spinning, eight silkworms were randomly selected and silk fibres were reeled off at 40 rpm; the first 500 m were discarded, and 50 m of fibre were degummed twice in a 0.5% NaHCO_3_ (wt %) solution at 100 °C for 30 min. The silk was washed and air-dried at room temperature and cut to lengths of 16 mm; the diameter of 30 randomly selected segments was measured by optical microscope (JEOL, Tokyo, Japan) (n = 3). The FTIR microspectroscopy procedures and the analysis of mechanical properties were carried out as previously described^40^ (Breaking strain describes the plastic properties of materials. Material fracture after total deformation, ΔL percentage of the original gauge length (L) ratio; Young’s modulus: describes the material elastically deformed. The larger the value, the more rigid is the material).

### Detection of flavonoids in sericin by HPLC

Eight cocoons were randomly selected and cut into pieces; 0.5 g of cocoon tissue was added to 200 ml 0.5% NaHCO_3_ (wt %) solution at 100 °C for 30 min, and then cooled to room temperature before centrifugation at 10,000 × g at 25 °C for 30 min. The flavonoid content of the supernatant was determined by HPLC using a C18 column (250 mm × 4.6 mm inner diameter, 5-μm particles) at 35 °C. The mobile phase was acetonitrile and 0.1% formic acid (75:25, v/v); injection volume was 10 μl; flow rate was 0.4 ml/min; and detection wavelength was 330 nm.

### Detection of EGT expression in hybrid offspring of Gal4 and UAS transgenic silkworms

#### Reverse transcription PCR

Fat bodies were dissected from silkworms (n = 15 per group) and placed in sterile polyethylene tubes. Total RNA was extracted using TRIzol reagent (Invitrogen, Carlsbad, CA, USA) and Moloney murine leukaemia virus reverse transcriptase (Invitrogen) was used to generate first-strand cDNA according to the manufacturer’s instructions. PCR amplification was carried under the following conditions: 95 °C for 30 s, and 25 cycles of 95 °C for 30 s and 60 °C for 30 s. *BmActin3* was used as a control. Primer sequences are listed in Table 1. Three independent replicates were analysed.

**Table 1:** Primers used in this study.

Gene	Genbank Accession No.	5′-3′	Purpose	Sequence	Remark
*GAL4*	AB186055.1	F	RT-PCR	GCAATCCCATTACCGCATATC	431 bp
		R		AGGTTCGGACCGTTGCTACTGT	
*EGT*	AY048771.1	F		CCAGCGTCCAGTATCTTG	649 bp
		R		CGACGACTGATGTCATGTC	
*BmActin3*	AB701689.1	F		AACACCCCGTCCTGCTCACTG	675 bp
		R		GGGCGAGACGTGTGATTTCCT	
*BmLP3*	FJ214661.1	F	For vector construction in transgenic larvae	GCgtcgacAGTATAGTTACAACGGCTGCCCC	1124 bp
		R		CGggatccGGAGCGTCGAGTCCTGCAATATG	
*GAL4*	AB186055.1	F		CGggatccATGAAGCTACTGTCTTCTATCG	2646 bp
		R		TTgcggccgcTTACTCTTTTTTTGGGTTTGGTGG	

Lower case letters indicate restriction sites.

### **SDS-PAGE and western blotting**

Total protein was quantified using a bicinchoninic reagent kit (Invitrogen) and 40 μg per sample were resolved by SDS-PAGE on 8% and 3–12% (w/v) polyacrylamide gradient gels for detection of EGT and sericin, respectively, according to the method of Laemmli^41^. Proteins were electrophoretically transferred to a polyvinylidene difluoride membrane (Roche Diagnostics, Indianapolis, IN, USA) that was probed with anti-EGT antibody (EGT protein was expressed in prokaryotic cells and provided to immunized rabbit to obtain EGT-polyclonal antibody) diluted 1:5000 in Tris-buffered saline with Tween 20 (10 mmol/l Tris-HCl, [pH 7.5], 150 mmol/l NaCl, and 0.05% Tween 20) containing 1% (w/v) bovine serum albumin. Horseradish peroxidase-conjugated goat anti-rabbit IgG (Beyotime Institute of Biotechnology, Shanghai, China) diluted 1: 10,000 in blocking buffer was used as the secondary antibody. Immunocomplexes were detected by enhanced chemiluminescence (Thermo Fisher Scientific, Waltham, MA, USA) and blots were imaged using a Clinx ChemiScope 3400 Mini (Science Instruments Co., Beijing, China).

### Treatment of larvae with 20E

On day 7 of the last-instar larval stage, 20E was sprayed on mulberry leaves fed to larvae at a dose of 1 μg/day/g body weight until they stopped eating; dimethylsulfoxide was used as a control. Each group contained 60 larvae. To determine the amount of 20E sprayed onto mulberry leaves, silkworms within each group were weighed. Briefly, if the total weight of the 60 larvae was 60 g, we would dissolve 60 μg 20E in 500 μl sterile water, and then spray this solution onto appropriate amount of mulberry leaves. These leaves were mixed with 20E and they were fed to silkworms when the solution on the leaves was dry. When silkworms finished these treated mulberry leaves, they were fed on normal mulberry leaves.

### Measurement of 20E level

Haemolymph was taken from the silkworm removing a proleg collected in a sterile polyethylene tube, and then centrifuged at 5000 × *g* for 10 min at 4 °C. To prevent melanisation of haemolymph, phenylthiourea was added to the supernatant at a final concentration of 2 mmol/l. Hemolymph samples from 15 silkworms were pooled. The concentration of 20E was measured as previously described^42^.

### Statistical analysis

All values are the mean ± SD, differences between groups were analysed with Student’s t-tests, considering P < 0.05 as the significance threshold. Single asterisk and double asterisks presented in figures indicate significant differences between treated and control groups at P < 0.05 and P < 0.01, respectively, and NS indicates no significant difference (p > 0.05).

## Electronic supplementary material

Supplementary Info
