# Supplementary material for: The expression of ecdysteroid UDP-glucosyltransferase enhances cocoon shell ratio by reducing ecdysteroid titre in last-instar larvae of silkworm, Bombyx mori
Source: Sci Rep. 2018 Dec 7;8:17710. doi: 10.1038/s41598-018-36261-y (PMC6286362; doi:10.1038/s41598-018-36261-y)
Supplement: Supplementary file 1 — Supplementary Info [file 41598_2018_36261_MOESM1_ESM.pdf]

Supplementary Information

The expression of ecdysteroid UDP-glucosyltransferase enhances cocoon shell ratio by reducing ecdysteroid titre in last-instar larvae of silkworm, *Bombyx mori*

Guanwang Shen[1,2], Jinxin Wu[1], Yong Wang[3], Hongling Liu[1], Haiyan Zhang[1], Sanyuan Ma[1], Chuyue Peng[3], Ying Lin[1,2], Qingyou Xia[1,2,*]

[1]State Key Laboratory of Silkworm Genome Biology, Southwest University, Chongqing 400716, China

[2]Chongqing Engineering and Technology Research Center for Novel Silk Materials, Chongqing 400716, China

[3]College of Biotechnology in Southwest University, Chongqing 400716, China

[*]Corresponding author: Qing You Xia, Professor

E-mail: xiaqy@swu.edu.cn

Address: 2 Tiansheng Rd, Beibei Qu, Chongqing 400716, China

Phone: 86-023-68250099

Fax: 86-023-68251288

E-mail: xiaqy@swu.edu.cn

Fig S1

A

B

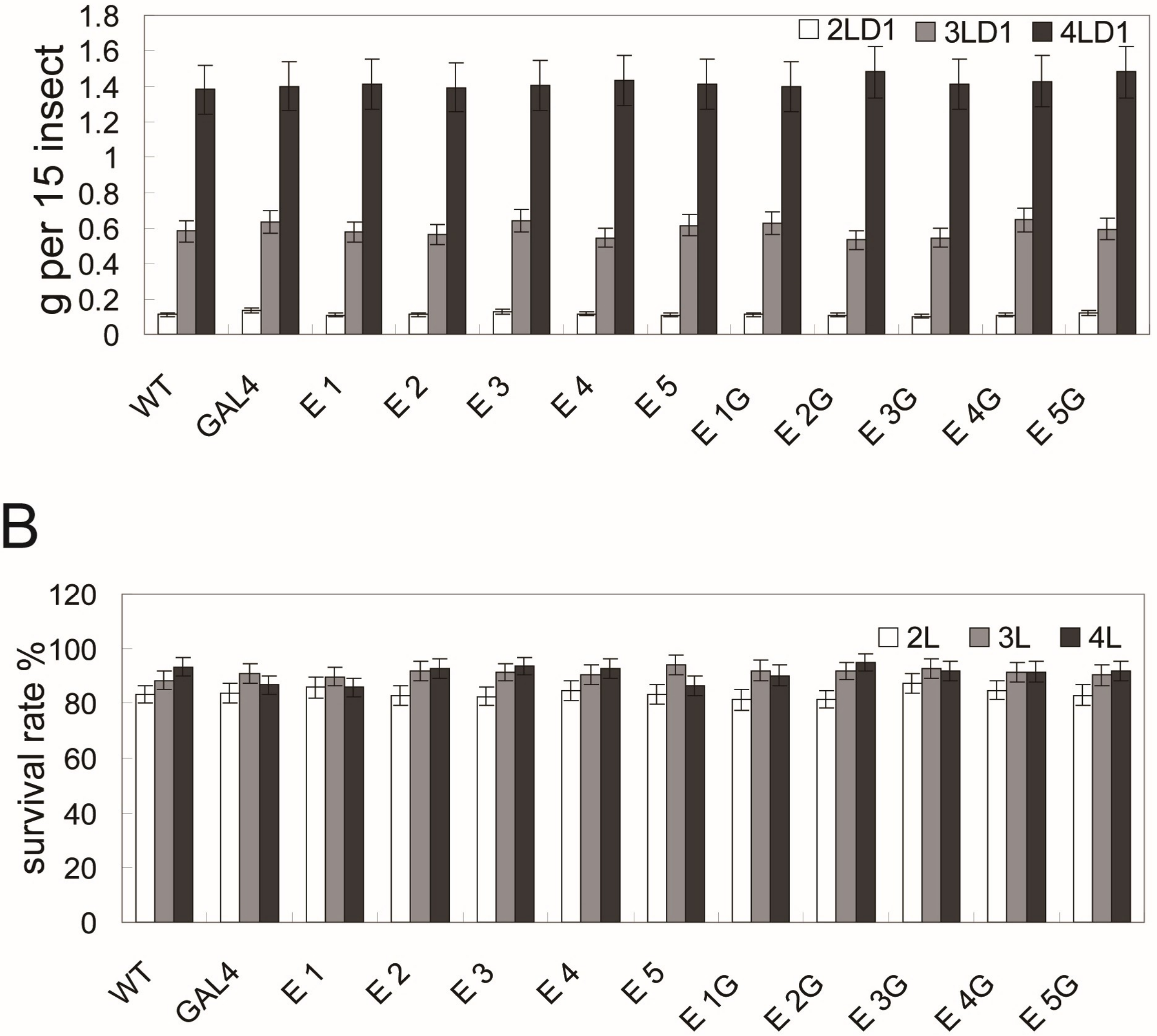

The larval body weight (Fig S1A) and survival rate percentage (Fig S1B) as Mean ± SD for wild-type (WT), non-hybrid transgenic (GAL4, E1-E5) and hybrid transgenic (E1G-E5G) silkworm larvae.
2LD1, 3LD1, and 4LD1 represent day 1 larvae of 2nd, 3rd and 4th instar larvae respectively.

Fig S2

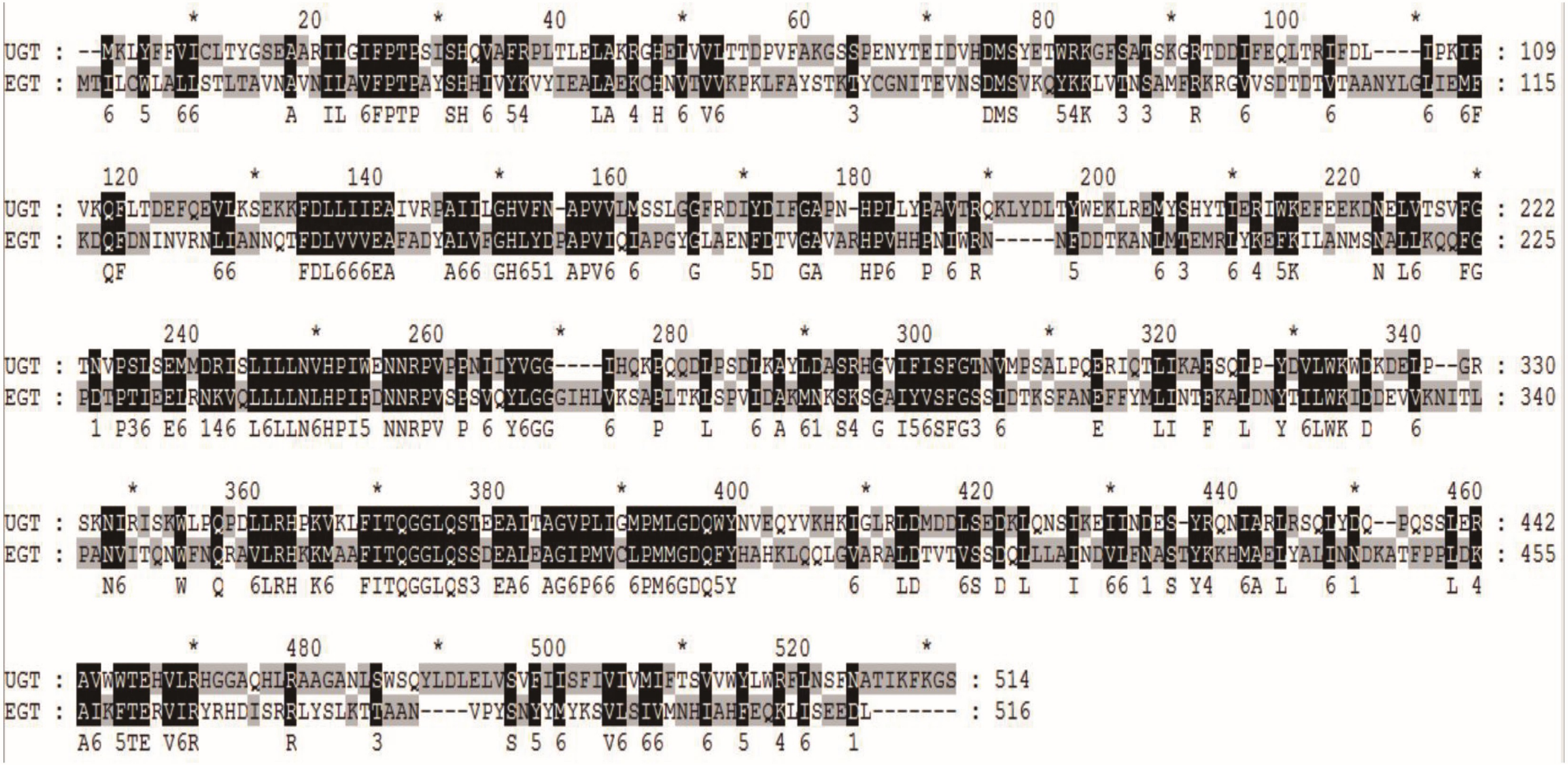

Amino acid sequence alignments of EGT and UGT

Fig S3

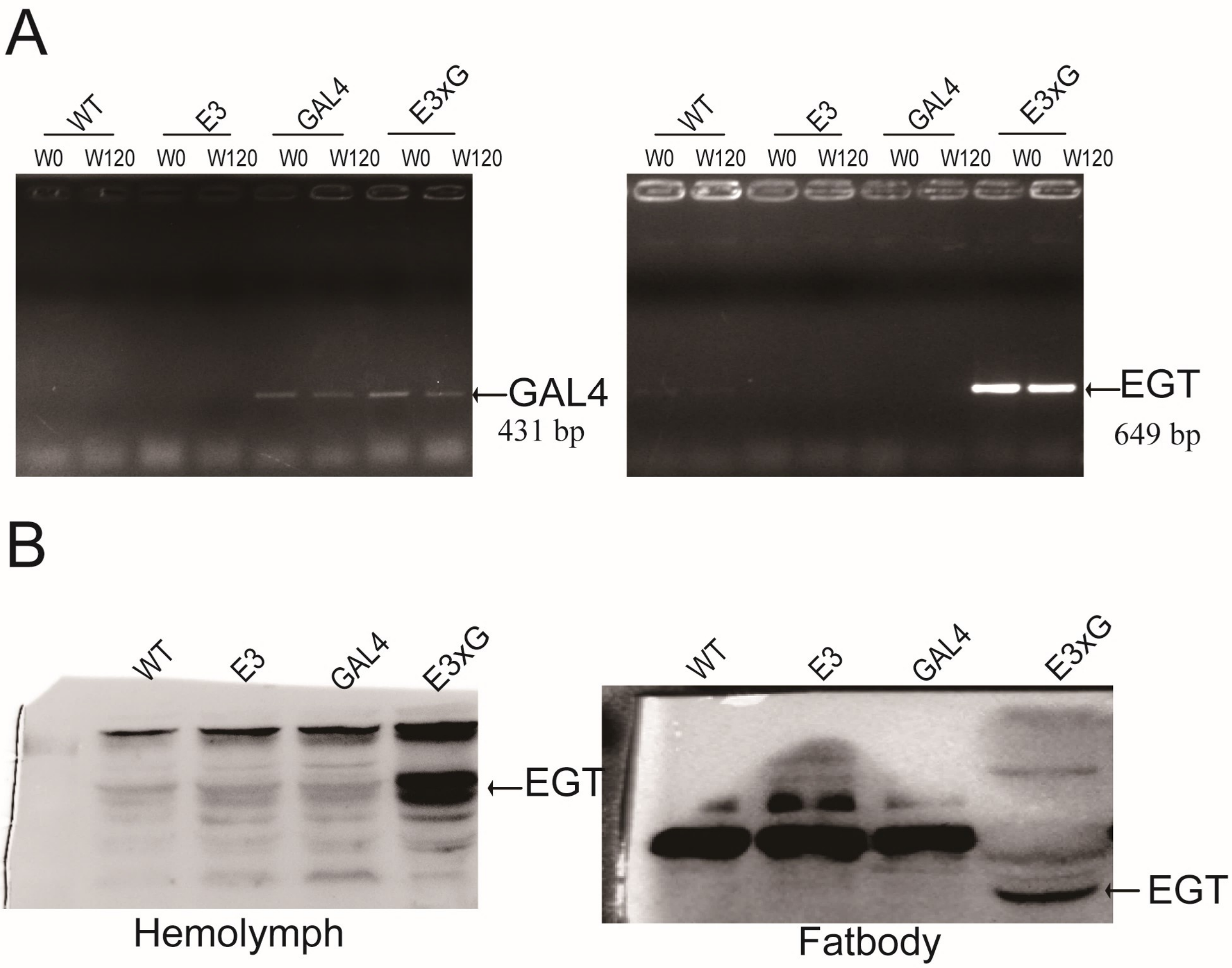

Fig S3 A. GAL4 and EGT mRNA levels in last-instar larval fat bodies at day 0 and day 5 after wandering
(original picture of Figure 3B)

Fig S3 B. EGT protein level on day 3 of wandering in last-instar larvae.
(original picture of Figure 3C)
